# DHA Attenuates Hypoxia/Reoxygenation Injury by Activating SSeCKS in Human Cerebrovascular Pericytes

**DOI:** 10.1007/s11064-019-02915-0

**Published:** 2019-11-27

**Authors:** Yanli Yu, Haibin Fang, Zhen Qiu, Zhongyuan Xia, Bin Zhou

**Affiliations:** Department of Anesthesiology, Renmin Hospital of Wuhan University, Wuhan, 430060 Hubei China

**Keywords:** Docosahexaenoic acid, SSeCKS, Ang-1/Ang-2, Hypoxia/reoxygenation injury, Human cerebrovascular pericytes

## Abstract

Docosahexaenoic acid (DHA) can alleviate cerebral ischemia/reperfusion injury by reducing blood–brain barrier permeability and maintaining its integrity, accompanied by an increased Ang-1/Ang-2 ratio; however, the underlying mechanisms of these effects remain unclear. Src-suppressed C kinase substrates (SSeCKS), a substrate of protein kinase C, plays an important role in maintaining cell junctions and cell morphology and regulating cell permeability. However, whether DHA can increase SSeCKS expression and then mediate the Ang-1/Ang-2 ratio still needs to be studied. Human cerebrovascular pericytes (HBVPs) cultured in vitro were divided into groups, treated with or without DHA along with SSeCKS siRNA to knockdown SSeCKS expression, and then subjected to 24 h of hypoxia followed by 6 h of reoxygenation. Cell viability; lactate dehydrogenase (LDH) release; and Ang-1, Ang-2 and VEGF activity were detected by using ELISA kits. The apoptosis rate was assessed by TUNEL flow cytometry. Expression of the SSeCKS, Ang-1, Ang-2 and VEGF proteins was evaluated by western blotting. Pretreatment with 10 μM or 40 μM DHA efficiently attenuated hypoxia/reoxygenation (H/R) injury by activating SSeCKS to increase the Ang-1/Ang-2 ratio and downregulate VEGF expression in HBVPs, as evidenced by decreased LDH release and apoptotic rates and increased HBVPs viability. Meanwhile, after we used SSeCKS siRNA to knock down SSeCKS protein expression, the protective effect of DHA on HBVPs following H/R injury was reversed. In conclusion, DHA can activate SSeCKS to increase the Ang-1/Ang-2 ratio and downregulate VEGF expression in HBVPs, thus reducing H/R injury.

## Introduction

Secondary injuries caused by traumatic brain injury and subarachnoid hemorrhage, such as cerebral ischemia, vasospasm and cerebral edema, are important factors in determining the recovery of neurological function and survival outcome [1]. Ischemic stroke is the sudden onset of cerebral blood circulation disorder and a pathological condition characterized by an initial restriction of blood to the brain [2]. Two major approaches have been developed to treat ischemic stroke: neuroprotection and reperfusion. Recanalization therapy provokes cerebral ischemia/reperfusion injury and impairs brain homeostasiss, which increases vascular permeability, disrupts the blood–brain barrier, and causes brain edema [3, 4]. The brain microvasculature, which is composed of endothelial cells, astrocytes, and peripheral cells, is an important part of the blood–brain barrier. Permeability of the damaged microvasculature, which results in cerebral hypoxia/reoxygenation (H/R) injury.

The vascular endothelial growth factor (VEGF) and angiopoietin-1 (Ang-1)/Tie-2 signaling pathways are closely related to continuous changes in microcirculation and barrier function after secondary injury [5]. VEGF is primarily responsible for the early promotion of vascular network formation, and plays an important role in protecting cerebral endothelial cells from H/R-induced injury. The combination of Ang-1 and Tie-2 can promote the stability of the vessel wall. Ang-2 is an antagonist of Ang-1, and the early and direct administration of Ang-1 or induction of Ang-1 expression was found to reverse imbalance in the Ang-1/Ang-2 ratio in a middle cerebral artery ligation mouse model [6]. This reversal could be achieved with the following mechanisms: the first mechanism was the reduction of Ang-2 release from Weibel-Palade bodies by pretreatment with statins [7], the second mechanism was the reduction of Ang-2 expression by nuclear transcription factor inhibitors [8], the third mechanism was the inhibition of Ang-2 by RNA oligonucleotide aptamers and blocking antibodies [9], and the fourth mechanism was the upregulation of Ang-1 expression in transfected carrier cells [10]. However, the clinical applications have not yet been reported.

Docosahexaenoic acid (DHA) is an essential, highly unsaturated fatty acid closely related to the normal function of cell membranes. DHA may be a promising drug for the treatment of cerebral ischemia/reperfusion injury [11]. Studies have reported that DHA can inhibit the expression of VEGF and its receptor, Flk-1, through the VEGF pathway to significantly inhibit the growth of corneal neovascularization and tumor blood vessels [12]. Recent studies have shown that DHA reduced apoptosis in rat brain microvascular endothelial cells induced by an underlying oxygen and glucose deprivation environment, thus decreasing Ang-2 and VEGF synthesis [13]. These results suggest that DHA may be a promising drug for cerebral ischemia injury related to angiogenin signaling pathway, but the specific regulatory mechanism of DHA is still unclear.

Src-suppressed C kinase substrates (SSeCKS), a substrate for protein kinase C and cytoskeletal cleavage factor, plays an important role in maintaining cell junctions and cell morphology and regulating cell permeability [14]. SSeCKS binds the intracellular portion of the transmembrane protein and regulates the stability of the blood–brain barrier through related signaling pathways. Studies have shown that hyperbaric oxygen exposure improved the permeability of the blood–brain barrier in rats with global cerebral ischemia injury with increased expression of caveolin-1 and tight junction proteins [15]. The expression of SSeCKS was decreased in astrocytes under hypoxic conditions and increased after oxygenation [16]. Furthermore, the high expression of SSeCKS in astrocytes downregulated VEGF expression and stimulated the secretion of Ang-1, increasing the Ang-1/Ang-2 ratio to reduce blood–brain barrier permeability and maintain its stability in human brain microvascular endothelial cells.

However, whether DHA can aggravate H/R-induced injury by activating SSeCKS experssion to increase the Ang-1/Ang-2 ratio and downregulate VEGF expression in human cerebrovascular pericytes (HBVPs) is unclear. The relationships between DHA and SSeCKS activation in H/R-induced HBVP injury and the SSeCKS-mediated Ang-1/Ang-2 ratio and VEGF expression under H/R stimulation are also unknown. In this study, we aimed to investigate the protective mechanism of DHA in ischemia/reperfusion injury with an in vitro H/R model in HBVPs.

## Materials and Methods

### Reagents

DHA (Beijing Zhongsha Jinqiao Biotechnology Co., Ltd); SSeCKS siRNA (Santa Cruz, CA, USA); ELISA kit and TUNEL kit (Jiancheng, Nanjing, China); Perkin Elmer Microplate reader (PerkinElmer Victor 1420, USA); Ang-1, Ang-2, and VEGF mouse anti-human monoclonal antibodies (R&D Systems, Minneapolis, USA); SSeCKS rabbit anti-human monoclonal antibodies (Santa Cruz, CA, USA); Goat anti-mouse polyclonal antibodies and mouse anti-rabbit polyclonal antibodies (R&D Systems, Minneapolis, USA); Odyssey color infrared laser scan-imaging instrument (Li-Cor, USA). FACSC calibur flow cytometer (BD, New Jersey, USA).

### Cell Culture

HBVPs isolated from abortion fetus human brain tissue were purchased from Shanghai Sangon Cell Center provided by ScienCell Research Laboratories. HBVPs was cultured in RPMI-1640 medium supplemented with 10% fetal bovine serum in an incubator at atmosphere of 5% CO_2_ and 37 °C. Medium was replaced every day and the cells were digested with 0.25% trypsin when the density of cells reached 80–90%. Passaged HBVPs cultured in vitro that were over 85% confluent were divided into the following experimental groups: ①control group (C); ②hypoxia/reoxygenation group (H/R); ③low dose DHA (10 μM) + H/R (LD + H/R); ④high dose DHA (40 μM) + H/R (HD + H/R). In order to evaluate whether SSeCKS knockdown reversed the protection of DHA by mediating the Ang-1/Ang-2 ratio and VEGF pathways in this process, the following experiments were performed: ⑤SSeCKS siRNA control group (SS); ⑥SSeCKS siRNA + H/R(SS + H/R); ⑦SSeCKS siRNA + H/R + LD(SS + LD + H/R); ⑧SSeCKS siRNA + H/R + HD(SS + HD + H/R). In DHA pretreatment groups, DHA (sigma, USA) was added to two final concentrations of 10 μM or 40 μM at a non-toxic concentration that had no effect on morphology or cell viability of HBVPs. SSeCKS siRNA or Scramble siRNA (Santa Cruz) was used to knockdown SSeCKS expression or as control following the manufacturer’s instructions. To test the effect of DHA alone on Ang-1, Ang-2 and VEGF protein levels, the low dose DHA (10 μM) or high dose DHA were also used under control conditions. The mixtures were given 1 h before hypoxia, then HBVPs were subjected to stimulated hypoxia for 24 h followed by 6 h of reoxygenation.

### Hypoxia/Reoxygenation Model

After pretreatment of each group, cells were then subjected to the model of H/R as described below. Cells cultured with RPMI-1640 medium without calf serum were subjected to a constant temperature three-gas incubator for 24 h to establish hypoxia in a condition with 94% N_2_, 5% CO_2_ and 1% O_2_ at 37 °C. Then, plates was replaced with RPMI-1640 supplemented with fetal calf serum at 37 °C and 5% CO_2_ chamber for 6 h to establish reoxygenation. In the pre-experiment, We explored the H/R-induced cell injury at different hypoxia or reoxygenation time points to determine the most suitable establishment of H/R injury model.

### Cell Viability Assay

Cell viability was determined by using a CCK-8 assay kit in 96-well plates. 10 μl of CCK-8 reagent was added to each well and then incubated for 3 h in darkness. The absorbance was detected at 450 nm using a Perkin Elmer Microplate Reader. The mean optical density (OD) of each group was used to calculate the percent of cell viability with the following formula: cell viability = treatment group OD/control group OD × 100%.

### LDH Activity Assay

LDH activity in the supernatants was measured for the evaluation of cell injury by using a commercially available LDH assay kit (Jiancheng, Nanjing, China) according to the manufacturer’s instructions.

### ELISA Analysis for Ang-1, Ang-2 and VEGF Level

The culture supernatants were collected after reoxygenation. The levels of Ang-1, Ang-2 and VEGF in supernatants were measured by using ELISA kits (Jiancheng, Nanjing, China) according to the manufacturer’s instructions.

### Apoptosis Assay

TUNEL flow cytometric assay was conducted to assess apoptosis. Apoptosis ratios of HBVPs under various treatments were measured by using Annexin V-fluorescein (AV) and propidium iodide (PI) apoptosis detection kit (Invitrogen, Carlsbad, CA, USA) by flow cytometry. Briefly, after stimulation, cells were washed with PBS and incubated with 10μL Annexin V-FITC and 5 μL PI for 15 min at room temperature in the dark. Flow cytometry analysis was done by FACS can flow cytometer (Beckman Coulter, Fullerton, CA, USA), and the data were analyzed by using Flow Jo software (Tree Star, Ashland, OR, USA).

### Western Blot Analysis

The levels of SSeCKS, Ang-1, Ang-2 and VEGF protein in cells were measured by western blotting. RIPA buffer was used to lyse and extract total protein from cells. Total protein (30 µg) was separated by 8% SDS-PAGE. Separated proteins were transferred to PVDF membranes under the influence of an electrical field. Primary monoclonal antibody was added (1:1000), followed by overnight incubation. Fluorescent secondary polyclonal antibody (1:15,000, CST, USA) was used for 1 h incubation at room temperature. The protein bands were detected with an odyssey color infrared laser scan-imaging instrument (Li-Cor, USA). The images were analyzed using odyssey Application Software 3.0.

### Statistical Analysis

All datas are represented as the mean ± SD. All statistical tests were performed by using GraphPad Prism version 6.0 (GraphPad Software, USA). One-way ANOVA followed by Tukey’s post hoc (a Bonferroni post hoc) test was performed to analyze the differences among experimental groups. *P* values < 0.05 was considered to be statistically significant.

## Results

### Hypoxia for 24 h and Reoxygenation for 6 h Significantly Reduced Cell Viability and Increased the Apoptosis Rate in Cultured HBVPs

**Fig. 1:**
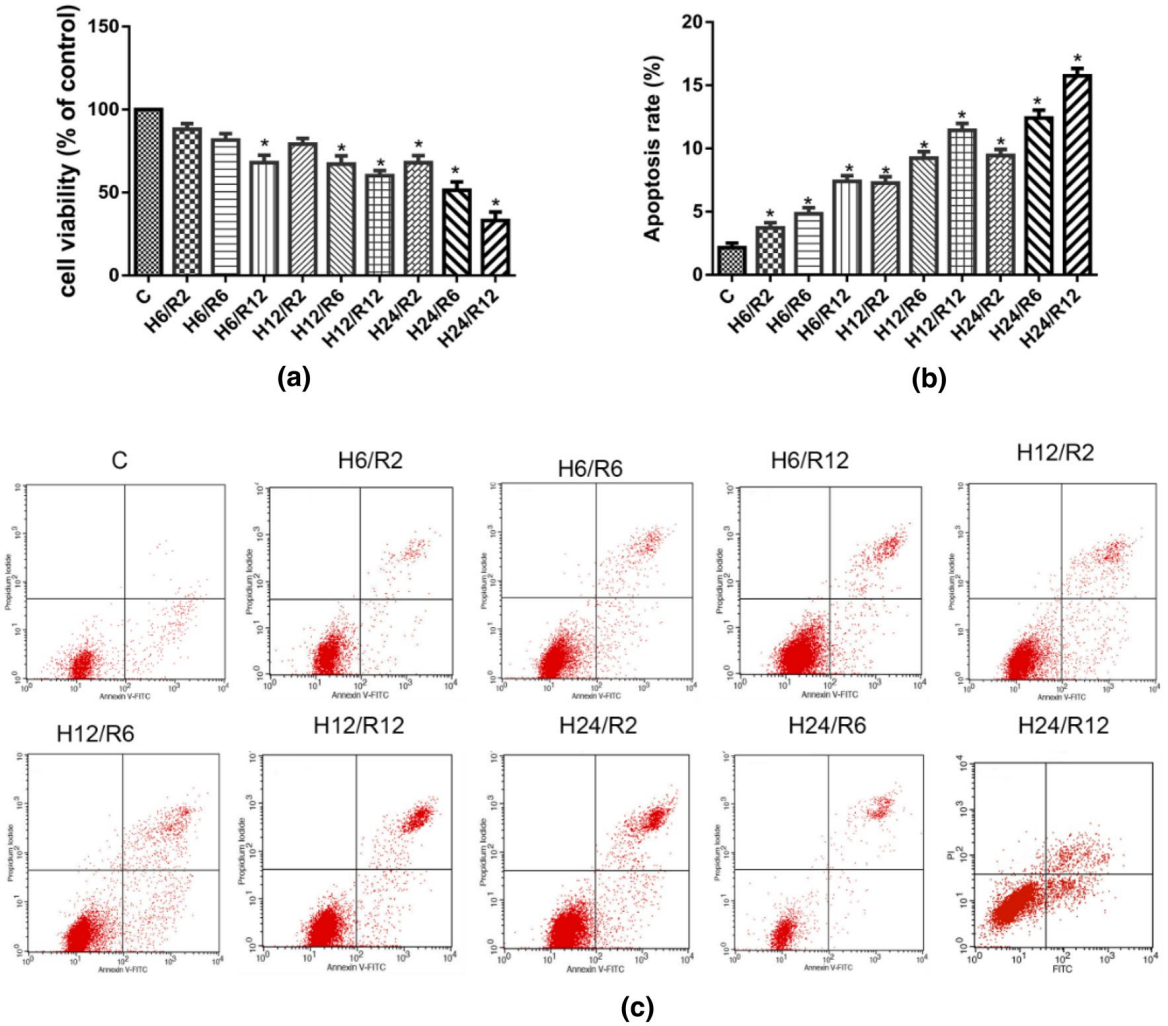
The cell viability and apoptosis rate of HBPVs following hypoxia/reoxygenation insult at different hypoxia and reoxygenation times. Data are expressed as mean ± SD (n = 6). ^*^P < 0.05 versus C group. *C* control, *H/R* hypoxia/reoxygenation

In the pre-experiment, we explored H/R-induced cell injury following hypoxia or reoxygenation for different lengths of time to confirm the establishment of H/R injury model. As shown in Fig. 1, compared with the control group, the degree of damage was gradually increased at 6 h, 12 h and 24 h of hypoxia and further aggravated with prolonged reoxygenation. As shown in Fig. 1a, the cell viability of HBVPs cultured under hypoxia for 24 h and reoxygenated for 6 h was significantly decreased compared with that of the control group. As shown in Fig. 1b, c, the apoptosis rate of the cells was significantly higher in the groups stimulated with H/R for different lengths of time than in the control group. After hypoxia for 24 h and reoxygenation for 6 h, the apoptosis rate increased significantly, and this difference was statistically significant. Thus, we chose hypoxia for 24 h and reoxygenation for 6 h as the most suitable H/R model for subsequent experiments.

### DHA Decreased Cell Injury and Apoptosis to Attenuated H/R Injury in HBVPs

**Fig. 2:**
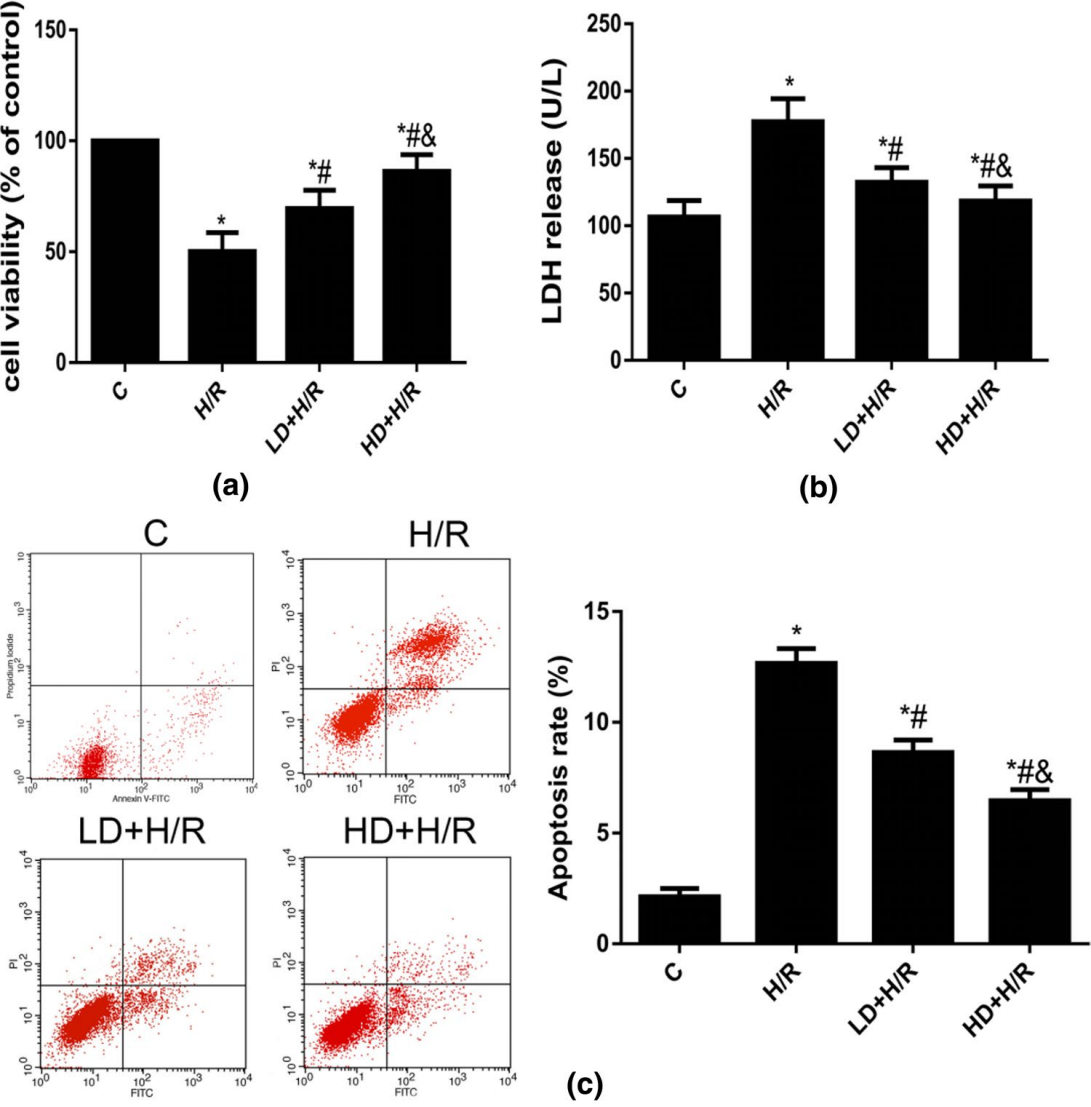
The effect of DHA on cell injury, LDH and appotosis rate after H/R insult at different concentrations in HBVPs. Data are expressed as mean ± SD (n = 6). ^*^P < 0.05 versus C group; ^#^*P* < 0.05 versus H/R group; ^&^*P* < 0.05 versus LD+H/R group.* C* control,* H/R* hypoxia/reoxygenation,* LD* low dose DHA,* HD* high dose DHA

To observe the effects of DHA on HBVP H/R injury by detecting cell viability, LDH release and apoptosis, HBVPs were exposed to H/R treatments along with 10 μM or 40 μM DHA. As shown in Fig. 2a, the cell viability in the H/R group was significantly decreased compared with that in the C group. LDH release and the apoptosis rate of the H/R group were also significantly increased compared with those of the control group (Fig. 2b, c). Compared with the H/R group, the 10 μM or 40 μM DHA significantly increased cell viability and decreased LDH release and the apoptosis rate in cultured HBVPs under H/R conditions (Fig. 2). Furthermore, DHA at the higher concentration tested (40 μM) further increased the cell viability and decreased LDH release and apoptosis rate compared to those in HBVPs treated with 10 μM DHA group (Fig. 2), indicating that DHA can significantly improve the cell survival rate and reduce apoptosis to protect HBVPs from H/R injury and that DHA at a high concentration had a more obvious protective effect than DHA at a low concentration.

### DHA Attenuated HBVP H/R Injury by Increasing the Ang-1 Level and Decreasing the Ang-2 and VEGF Levels in Culture Supernatants

**Fig. 3:**
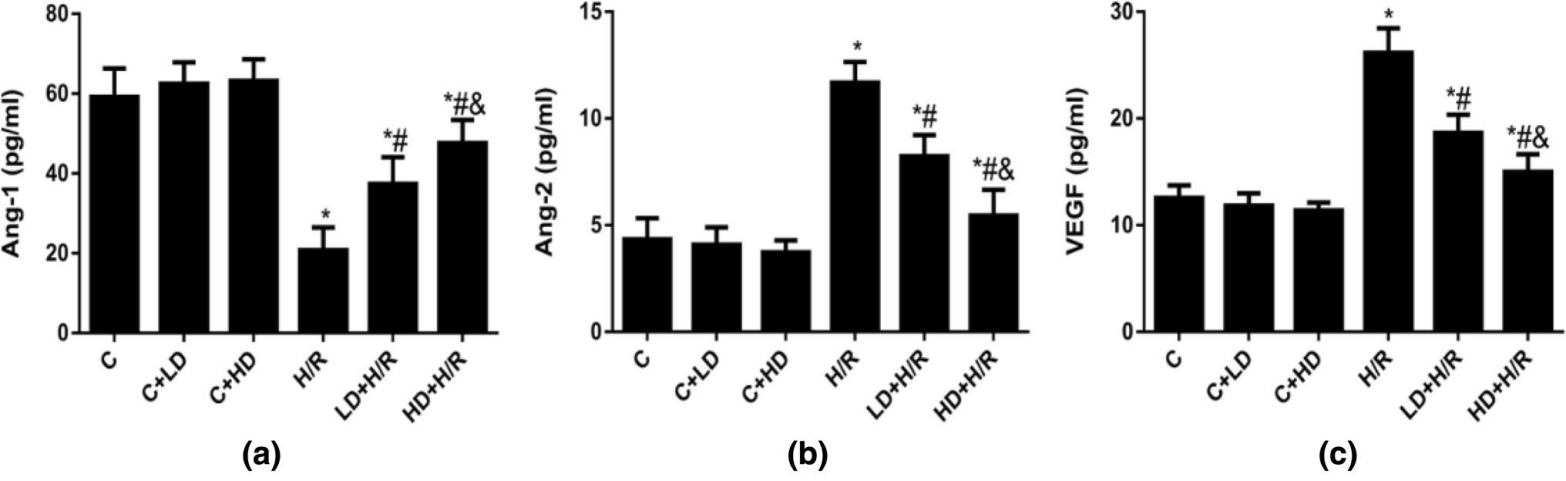
The effect of DHA on the protein levels of Ang-1, Ang-2 and VEGF in HBVP culture supernatants after H/R insult at different concentrations. Data are expressed as mean ± SD (n = 6). ^*^P < 0.05 versus C group; ^#^*P* < 0.05 versus H/R group; ^&^*P* < 0.05 versus LD + H/R group. *C* control, *H/R* hypoxia/reoxygenation, *LD* low dose DHA, *HD* high dose DHA.ata are expressed as mean ± SD (n = 6). ^*^P < 0.05 versus C group; ^#^*P* < 0.05 versus H/R group; ^&^*P* < 0.05 versus LD + H/R group. *C* control, *H/R* hypoxia/reoxygenation, *LD* low dose DHA, *HD* high dose DHA

Ang-1/Ang-2 plays important roles in blood–brain barrier permeability, endothelial cell fusion, and vascular reactivity to different organ systems and disease states [5, 6, 17]. To investigate the roles of the Ang-1/Ang-2 and VEGF signaling pathways in DHA-attenuated HBVP H/R injury, we next measured the levels of Ang-1, Ang-2 and VEGF in HBVP culture supernatants. The level of Ang-1 was decreased after H/R stimulation compared with that in the C group, and the levels of Ang-2 and VEGF were increased in the H/R group compared with the C group. As shown in Fig. 3a–c, the levels of Ang-1, Ang-2 and VEGF were not significantly changed after DHA treatment under control conditions. However, the levels of Ang-1 were higher in the LD + H/R and HD + H/R groups than in the H/R group, and the levels of Ang-2 and VEGF were lower in the LD + H/R and HD + H/R groups than in the H/R group (Fig. 3). Moreover, compared with 10 μM DHA treated group, the level of Ang-1 was higher in the 40 μM DHA treated group, accompanied by lower levels of Ang-2 and VEGF. These results indicated that DHA attenuated cell injury in HBVPs under H/R stimulation by increasing Ang-1/Ang-2 levels and decreasing VEGF levels.

### DHA Protects Against H/R Injury by Upregulating the Protein Expression of SSeCKS and Ang-1 and Decreasing the Expression of Ang-2 and VEGF in HBVPs

**Fig. 4:**
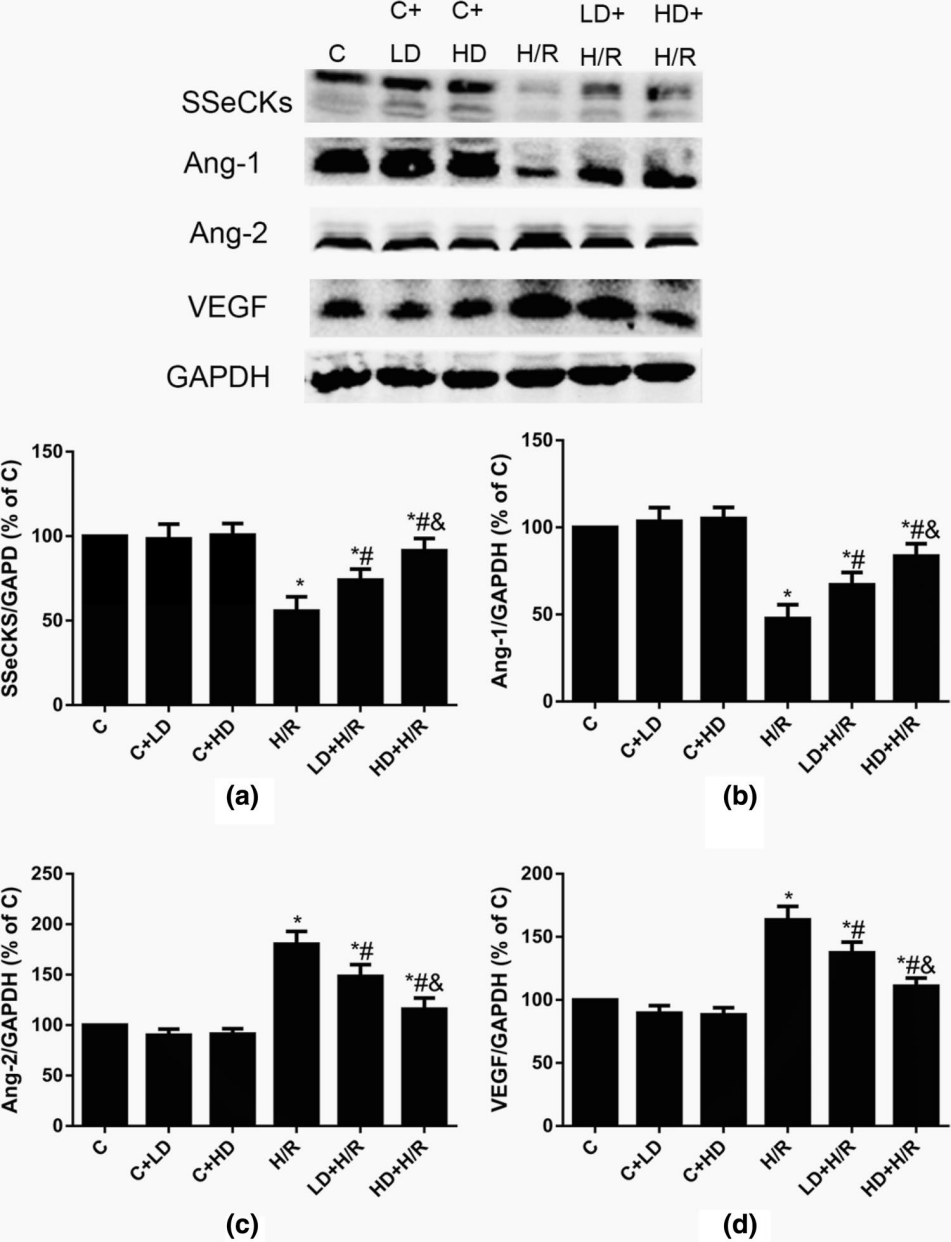
Effects of DHA on SSeCKS, Ang-1, Ang-2 and VEGF proteins expression in HBVPs after H/R insult at different concentration. Data are expressed as mean ± SD (n = 6). ^*^P < 0.05 versus C group; ^#^*P* < 0.05 versus H/R group; ^&^*P *< 0.05 versus LD + H/R group. *C* control, *H/R* hypoxia/reoxygenation, *LD* low dose DHA, *HD* high dose DHA

DHA can significantly reduce inflammation and tissue damage after traumatic spinal cord injury [18]. Studies have indicated that DHA can inhibit the expression of VEGF and its receptor, making DHA a promising drug for the treatment of ischemic brain injury [19]. SSeCKS expression has been reported to play a novel role in maintaining blood–brain barrier properties and increase the expression of tight junction proteins in the process of cerebral vascular differentiation. Furthermore, SSeCKS can increase Ang-1 expression to mediate cerebral ischemia injury [20]. Therefore, we next detected the expression of SSeCKS, Ang-1, Ang-2 and VEGF in HBVPs to explore the specific molecular mechanisms of these proteins. As shown in Fig. 4a, b, the levels of SSeCKS and Ang-1 were decreased after H/R stimulation compared with that in the C group. The protein levels of SSeCKS, Ang-1, Ang-2 and VEGF were not significantly changed after DHA treatment under control conditions as shown in Fig. 4. However, the levels of SSeCKS and Ang-1 in the LD + H/R and HD + H/R groups were significantly higher than those in the H/R group, and 40 μM DHA further increased these changes. As shown in Fig. 4c, d, the levels of Ang-2 and VEGF were increased in the H/R group compared with the C group. Compared with those in the H/R group, the levels of Ang-2 and VEGF were decreased in the groups treated with DHA at both a low and high dose, and higher DHA concentrations (40 μM) further decreased the levels of Ang-2 and VEGF compared with low DHA concentrations (10 μM). These results indicated that DHA may regulate the levels of angiogenic factors under hypoxic-ischemic conditions by regulating SSeCKS expression to protect HBVPs from H/R injury and that 40 μM DHA had a more obvious protective effect than 10 μM DHA.

### SSeCKS Gene Knockdown with siRNA Abrogated the Protective Effects of DHA in H/R Injury in Cultured HBVPs

**Fig. 5:**
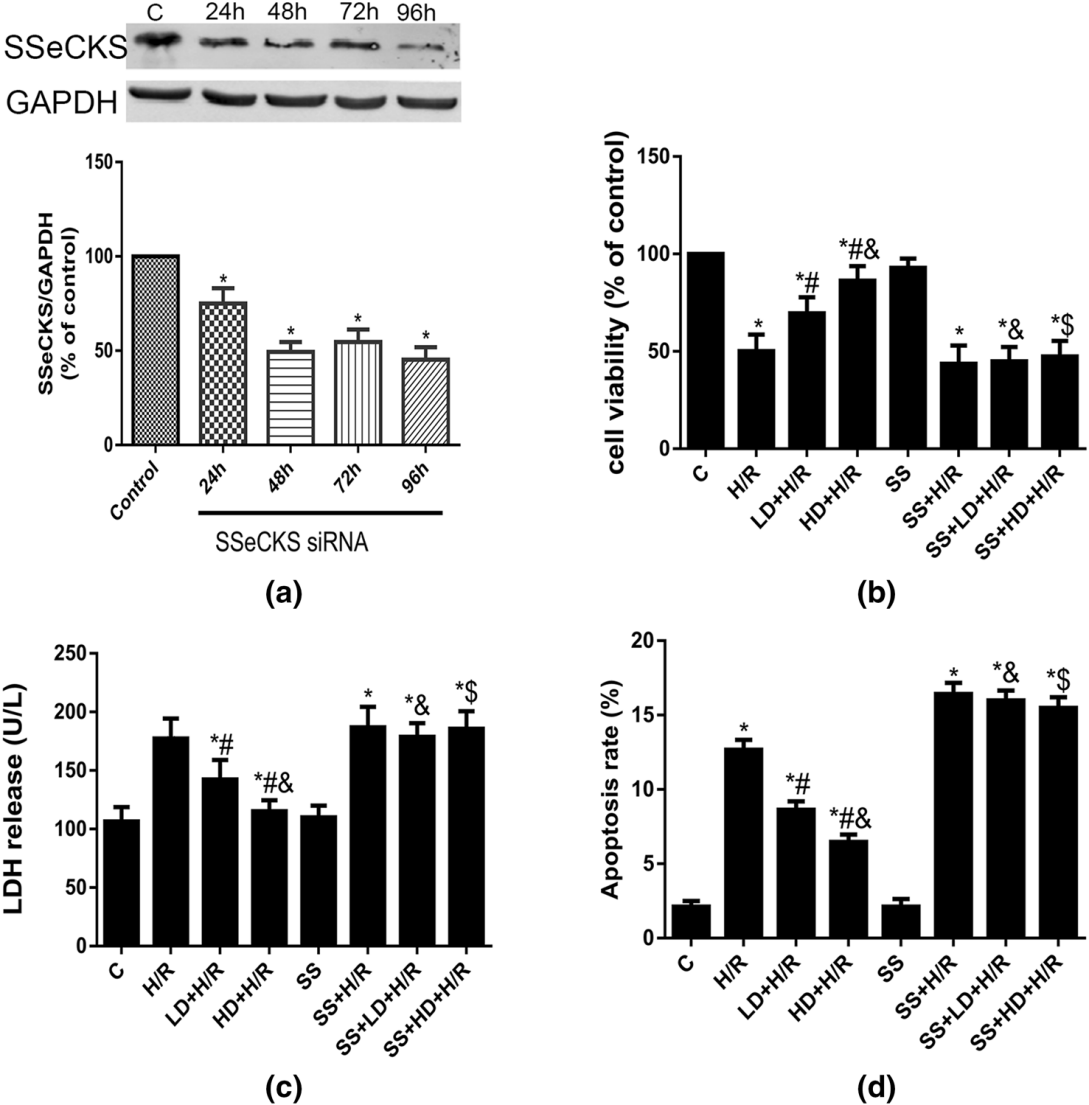
Effects of SSeCKS gene knockdown with siRNA on SSeCKS, cell viability, LDH release and apoptosis rate in HBVPs after H/R insult with or without DHA pretreatment. Data are expressed as mean ± SD (n = 6). ^*^P < 0.05 versus C group and/or SS group; ^#^*P* < 0.05 versus H/R group; ^&^*P *< 0.05 versus LD + H/R group; ^$^*P *< 0.05 versus HD + H/R group. *C* control, *H/R* hypoxia/reoxygenation, *LD* low dose DHA, *HD* high dose DHA, *SS* SSeCKS siRNA

To further confirm that the inhibition of SSeCKS can abrogate the protective effects of DHA in HBVPs with H/R injury, we knocked down SSeCKS expression in HBVPs with SSeCKS siRNA. As shown in Fig. 5a, after the transfection of SSeCKS siRNA for 12 h, the SSeCKS protein level decreased significantly, and SSeCKS protein expression was inhibited by close to 50% after SSeCKS siRNA transfection for 48 h. Therefore, in further treatments, we performed a subsequent experiment after 48 h of SSeCKS siRNA treatment. As shown in Fig. 5b–d, the cell viability, LDH release and apoptosis rate in the SS group were not significantly different than those in the C group; the cell viability of the SS + H/R group was significantly lower than that of the C and SS groups and accompanied by increased LDH release and apoptosis rates. Compared with those of the C and SS groups, the cell viabilities of the SS + H/R, SS + LD + H/R and SS + HD + H/R groups were significantly decreased, and LDH release and the apoptosis rate were significantly increased; but there were no differences in cell viability, LDH release, or apoptosis rate among the SS + H/R, SS + LD + H/R and SS + HD + H/R groups. Moreover, compared with that of the LD + H/R group, the cell viability of SS + LD + H/R groups was significant decreased, accompanied by increased LDH release and apoptosis rates. The same differences were observed between the HD + H/R and SS + HD + H/R groups. All of these results indicated that DHA pretreatment had no protective effect when SSeCKS expression was knocked down in HBVPs under H/R conditions.

### Effects of DHA on SSeCKS, Ang-1, Ang-2 and VEGF Expression in HBVPs with SSeCKS Knockdown Exposed to H/R

**Fig. 6:**
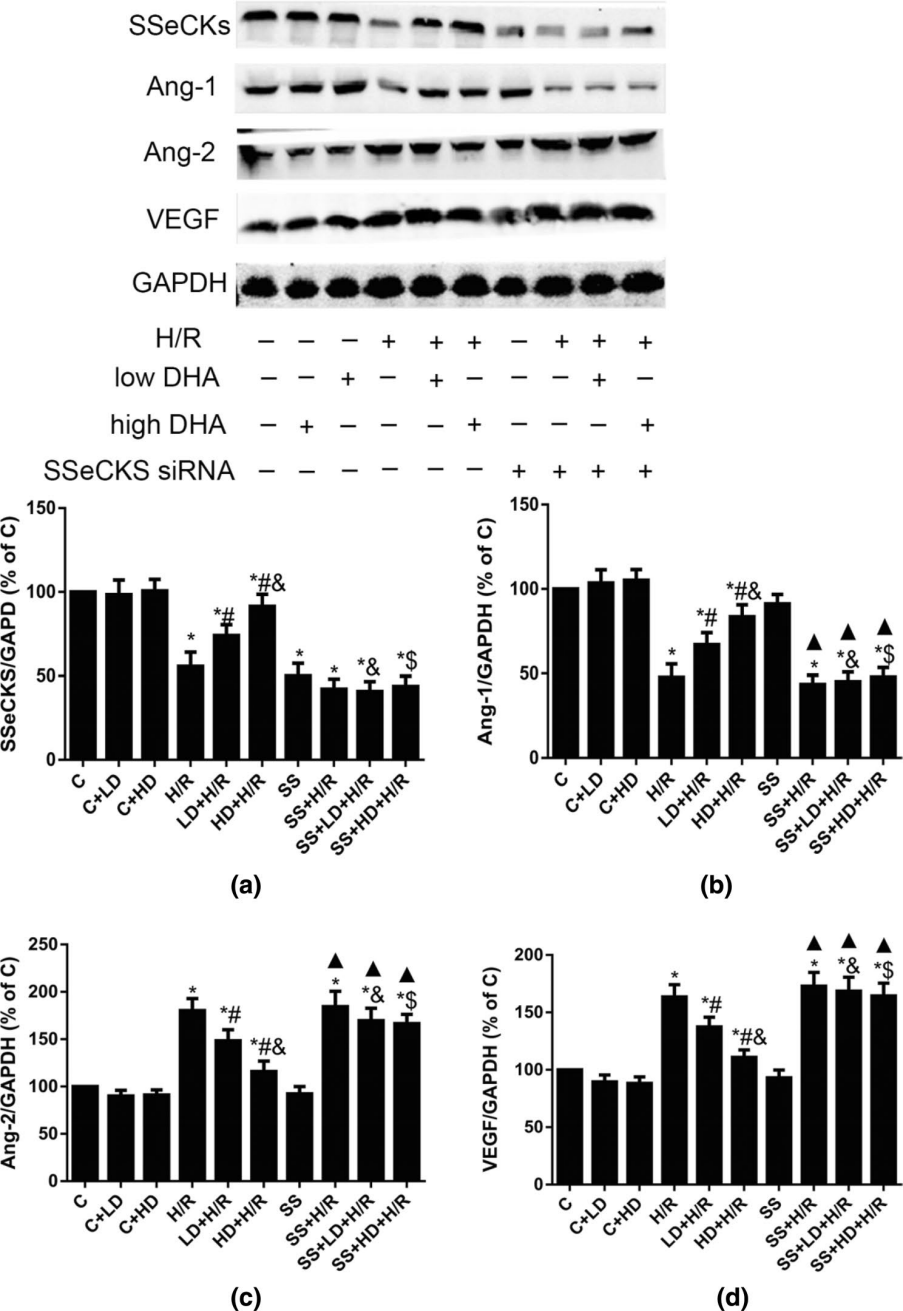
Effects of DHA with SSeCKS knockdown on SSeCKS, Ang-1, Ang-2 and VEGF expression in HBVPs exposed to H/R. Data are expressed as mean ± SD (n = 6). ^*^P < 0.05 versus C group; ^#^*P* < 0.05 versus H/R group; ^&^*P *< 0.05 versus LD + H/R group; ^▲^*P* < 0.05 versus SS group; ^$^*P *< 0.05 versus HD + H/R group. *C* control, *H/R* hypoxia/reoxygenation, *LD* low dose DHA, *HD* high dose DHA, *SS* SSeCKS siRNA

As shown in Fig. 6a, the SSeCKS protein expression in the SS, SS + H/R, SS + LD + H/R and SS + HD + H/R groups was significantly lower than that in the C group. However, there was no significant difference in SSeCKS expression among the SS, SS + H/R, SS + LD + H/R and SS + HD + H/R groups, indicating that DHA did not significantly alter SSeCKS expression following SSeCKS siRNA transfection. As shown in Fig. 6b–d, there was no difference in Ang-1, Ang-2 and VEGF expression between the SS and C groups. Compared with that in the C and SS groups, the Ang-1 protein level was significantly decreased and Ang-2 and VEGF were significantly upregulated in the SS + H/R, SS + LD + H/R and SS + HD + H/R groups. Compared with those in the SS + H/R group, the Ang-1, Ang-2 and VEGF protein levels in the SS + LD + H/R and SS + HD + H/R groups were not significantly different. Moreover, compared with that in the LD + H/R group, the Ang-1 expression was significantly decreased in the SS + LD + H/R, accompanied by increased Ang-2 and VEGF levels. The same differences were observed between the HD + H/R and SS + HD + H/R groups. These results suggested that the protective effect of DHA on HBVPs is achieved by upregulating SSeCKS.

## Discussion

Ischemic brain injury, also known as ischemic stroke, is the sudden onset of cerebral blood circulation disorders, including ischemic stroke (transient ischemic attack, atherothrombotic cerebral infarction, lacunar infarction, cerebral embolism), hemorrhagic stroke (cerebral hemorrhage, subarachnoid hemorrhage), high blood pressure encephalopathy and vascular dementia. During the rescue and treatment of ischemic diseases, the main factor that causes damage to tissues is not ischemia itself but rather the recovery of the blood supply; this damage is called ischemia/reperfusion injury. Studies have reported that cerebral ischemic/reperfusion injury causes blood–brain barrier disruption, which accelerates the development of abnormal vascular permeability and exacerbates brain edema [21]. It was reported that SSeCKS expression in hypoxic astrocytes was decreased, but increased after reoxygenation [16, 20]. This increase in SSeCKS inhibited angiogenesis and provided endothelial cells with properties of the blood–brain barrier, significantly reducing human brain microvascular endothelial cell migration and capillary-like structure formation. DHA has been suggested to play an important role in alleviating the pathogenesis of ischemic brain injury by attenuating edema and neuronal loss and improving synaptic connection [22]. However, the effect of DHA in H/R-stimulated HBVPs and the relationship between DHA and SSeCKS are unclear. In the present study, we first demonstrated that pretreatment with DHA at final concentrations of 40 μM and 10 μM significantly improved cell viability and decreased apoptosis to attenuate H/R injury in HBVPs by activating SSeCKS to mediate the Ang-1/Ang-2 and VEGF pathways. After SSeCKS gene silencing, the protective effects of DHA against H/R injury in cultured HBVPs were abrogated with downregulated Ang-1 levels and upregulated Ang-2 and VEGF expression. These results suggest that DHA can attenuate H/R injury in HBVPs, which may be achieved by upregulating SSeCKS, which then increases Ang-1 expression and decreases Ang-2 and VEGF expression. These findings indicate that DHA is a potential, novel, therapeutic target for cerebral ischemia/reperfusion injury.

The main pathophysiological changes in cerebral ischemia are the destruction of the blood–brain barrier and the entrance of macromolecular substances into the brain tissue, leading to cerebral edema and even bleeding [23]. Thrombolytic therapy allows the occluded blood vessels to be recanalized, and ischemic brain tissue is restored in time, but this restoration is accompanied by complex pathophysiological changes that aggravate cell damage and cell death and then induce cerebral ischemia/reperfusion injury [24]. Destruction of endothelial cells and microvascular structures leads to increased permeability of the blood–brain barrier and the extravasation of plasma macromolecules and harmful substances to aggravate cerebral edema after ischemic injury. Pericytes play an important role in the transport of blood–brain barrier substances and regulation of vascular permeability and promotes the formation of tight junctions of endothelial cells to maintain the structure and function stability of the blood–brain barrier [25, 26]. In this experiment, we established a model of H/R in HBVPs. The results showed that cell viability was significantly decreased and that LDH release and the apoptosis rate were significantly increased in HBVPs after hypoxia for 24 h and reoxygenation for 6 h. These findings emphasize that we successfully established an HBVP H/R model and that H/R can induce significant cell injury in HBVPs.

Ang and VEGF are two major proangiogenic factors that synergistically promote angiogenesis [27]. The expression level of VEGF is significantly increased in pathological conditions such as hypoxia and injury [28, 29]. Studies have shown that the expression of VEGF is significantly upregulated in H/R-induced rat brain endothelial cell injury [30], and that the peak level of VEGF expression is related to the severity of brain injury. Angiopoietins are proteins secreted by vascular endothelial cells, pericytes, myocytes, and mesenchymal cells. The competitive binding of Ang-1 and Ang-2 to Tie-2 plays an important role in blood–brain barrier permeability, endothelial cell fusion, and the response of blood vessels to different organ systems and disease states [31–33]. Further studies showed that downregulated Ang-1 expression is closely related to blood–brain barrier function after cerebral ischemia and that the administration of Ang-1 can alleviate injury to the blood–brain barrier induced by ischemia, while Ang-2 and VEGF cause damage blood–brain barrier permeability [34, 35]. After cerebral ischemia, the expression of VEGF and Ang-2 is rapidly upregulated, resulting in an imbalance of the Ang-2/Ang-1 ratio and increased blood brain barrier permeability [36, 37]. Pericytes embrace the capillary endothelium, contributing to the maturation of vessels. Pericyte manifestations are related to changes in blood–brain barrier permeability by an increase in endocytosis-mediated transport and tight junction disruption. Ang-1 secreted by brain astrocytes and pericytes contributes to formation of the blood–brain barrier, whereas Ang-2 directly breaks down the blood–brain barrier [38]. In this study, we found that H/R could induce significant cell injury in HBVPs with the increased secretion of Ang-2 and VEGF and decreased Ang-1 secretion in the supernatants. The decreased expression of Ang-1 and increased expression of Ang-2 and VEGF in tissue also confirmed this trend. It has been speculated that oxygen–glucose–deprivation conditions cause early hypoxia, hypoglycemia damage and subsequent reoxygenation injury by changing the corresponding angiopoietin and VEGF protein pathways, resulting in a steady-state imbalance in the surrounding environment.

As an important component of polyunsaturated fatty acids, DHA is an essential highly unsaturated fatty acid in the human body that is closely related to the normal function of cell membrane molecules. DHA is widely distributed in the central nervous system and plays an important role in maintaining the integrity of neurons and the transmission of information. Quartu et al. [4] reported that the administration of frankincense essential oils (unsaturated fatty acids containing DHA and eicosapentaenoic acid) before cerebral ischemia can reduce the inflammatory response of brain tissue by downregulating the expression of COX-2 and provide nutritional support to damaged brain tissue, improving the cell survival rate and thus reducing cerebral ischemia/reperfusion injury. Paterniti et al. [31] confirmed that DHA can significantly reduce inflammation and tissue damage after traumatic spinal cord injury. Further studies have shown that DHA can alleviate cerebral ischemia/reperfusion injury, maintain blood–brain barrier integrity, and reduce blood–brain barrier permeability [5]. Das S et al. reported that DHA can inhibit the expression of VEGF and its receptor, Flk-1, through the VEGF pathway, thereby significantly inhibiting corneal neovascularization and tumor vascular growth [12]. On the other hand, eicosapentaenoic acid could effectively reduce the level of Ang-2 in the plasma of chronic diabetic patients [39]. Therefore, DHA has important research significance and clinical prospects as a new drug to alleviate brain damage. In this study, we utilized DHA at final concentrations of 10 μM or 40 μM to stimulate HBVPs under H/R conditions. Our preliminary experiments indicated that the protein levels of Ang-1, Ang-2 and VEGF were no significantly changed after DHA treatment under normal conditions. However, after H/R stimulation, 10 μM or 40 μM DHA sinificantly increased cell viability and decreased apoptosis by upregulating the expression of SSeCKS and Ang-1 and downregulating the expression of Ang-2 and VEGF. These results indicated that DHA may regulate the levels of angiogenic factors under hypoxic-ischemic conditions by regulating SSeCKS activation to protect HBVPs from H/R injury.

**Fig. 7:**
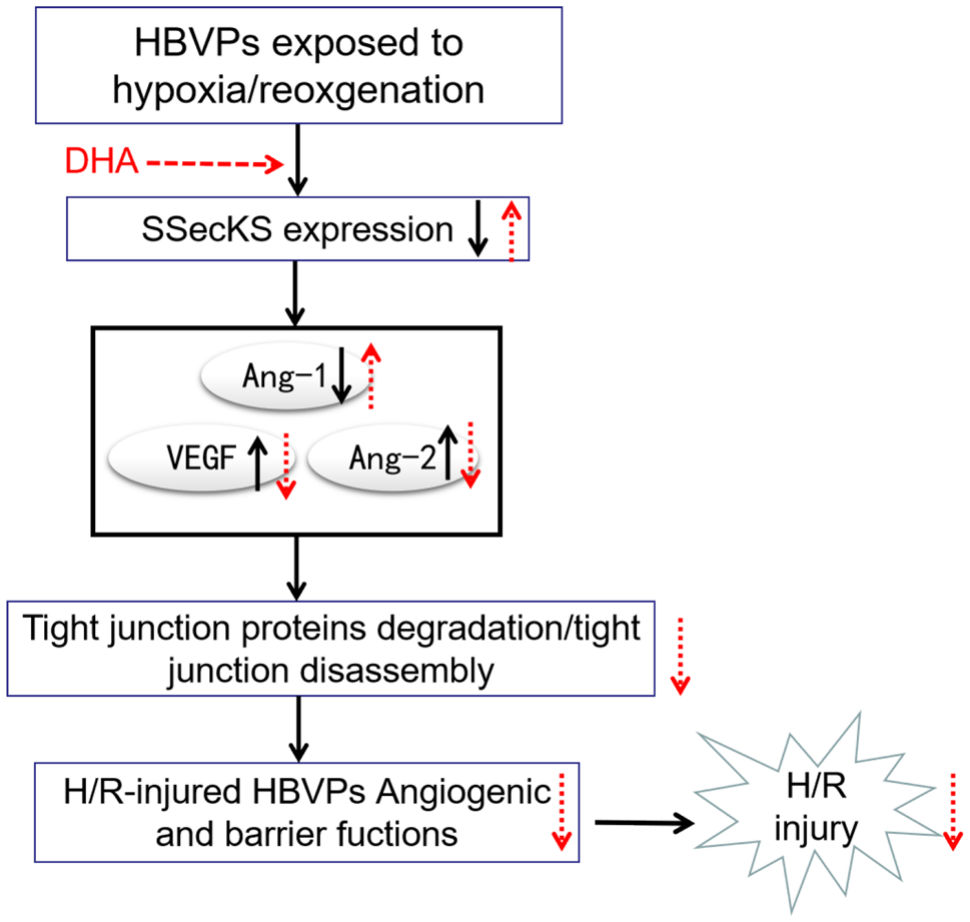
The mechanisms underlying the protection of DHA against HBVPs injury induced by hypoxia/reoxygenation (H/R). Briefly, hypoxia/reoxygenation resulted in decrease in SSeCKS, then decreasing Ang-1 and increasing Ang-2 and VEGF which degraded the tight junction proteins or led to tight junction disassembly, further causing changes in HBVPs angiogenic and barrier fuctions. Eventually, these alterations led to H/R injury. Interestingly, DHA pretreatment notably up-regulated the expression levels of SSeCKS, then increasing Ang-1 and decreasing Ang-2 and VEGF, further enhanced the tight junction proteins and barrier fuctions changes. As a result, DHA administration inhibited H/R-induced injury in HBVPs. The open arrow indicate the effects of DHA pretreatment on HBVPs injury following hypoxia and reoxygenation

SSeCKS is a substrate of protein kinase C and a cytoskeletal cleavage factor. It plays an important role in maintaining cell junctions and cell morphology, regulating cell permeability, promoting blood–brain barrier maturation and maintaining blood–brain barrier integrity [14]. The expression of SSeCKS was decreased in astrocytes under hypoxic conditions but increased after oxygenation [16]. The expression of high levels of SSeCKS downregulated the expression of VEGF and upregulated the secretion of Ang-1 to increase the Ang-1/Ang-2 ratio by increasing the expression of tight junction proteins such as band-like cloning protein 1 (ZO-1), reduce blood–brain barrier permeability and maintain the blood–brain barrier in HBVPs [14, 20]. SSeCKS reduced the expression of vascular endothelial growth factor by reducing the activity of the transcription factor AP-1 [14]. In the process of cerebral vascular differentiation, SSeCKS increased the expression of tight junction proteins and gradually transformed the permeable capillary plexus into a vascular plexus with blood–brain barrier properties. In our present study, we found that the expression of SSeCKS protein was significantly decreased after H/R stimulation in HBVPs, which is consistent with the results of a study by Lee et al. [14]. When we used DHA to pretreat the cells, H/R injury was significantly reduced, which was accompanied by significantly increased SSeCKS expression, an increased Ang-1/Ang-2 ratio and decreased VEGF expression. Furthermore, when we used SSeCKS siRNA to knock down the SSeCKS gene, the protective effect of DHA in HBVPs under H/R insult disappeared with a significant change in the Ang-1/Ang-2 ratio and VEGF expression. Taken together, our results demonstrated that the protective effect of DHA on HBVP H/R injury may be achieved by upregulating the activation of SSeCKS to increase the expression of Ang-1 and decrease the levels of Ang-2 and VEGF (Fig. 7).

In conclusion, our findings suggested that H/R insult can significantly induce HBVP injury by decreasing the levels of SSeCKS and Ang-1 and increasing the expression of Ang-2 and VEGF. DHA could alleviate H/R injury in HBVPs by increasing Ang-1 expression and the Ang-1/Ang-2 ratio and decreasing VEGF expression, which may be related to an increase in SSeCKS activation, and may be a new therapeutic target to mitigate cerebral ischemia/reperfusion injury. However, the specific mechanisms of DHA-mediated SSeCKS activation and alteration of the Ang-1/Ang-2 ratio and VEGF levels to alleviate H/R injury remain to be further addressed.
